# Guidewire-assisted en bloc resection of periorifice non-muscle-invasive bladder cancer: a retrospective case series

**DOI:** 10.1186/s12894-026-02279-y

**Published:** 2026-07-25

**Authors:** Qiu Yao, Hui Niu, Huizhong Jiang, Lijun Xu, Yachen Zang, Wei Wu, Jin Zhu, Boxin Xue

**Affiliations:** 1 Department of Urology, The Second Affiliated Hospital of Soochow University, 1055 Sanxiang Road, Suzhou, Jiangsu 215004 China; 2 Department of Pathology, The Second Affiliated Hospital of Soochow University, Suzhou, Jiangsu 215004 China; 3 Department of Operating Room, The Second Affiliated Hospital of Soochow University, Suzhou, Jiangsu 215004 China; 4 Department of General Medicine, Linhu Town Health Center, Suzhou, Jiangsu 215004 China

**Keywords:** En bloc resection of bladder tumor, Holmium laser, Non-muscle-invasive bladder cancer, Ureteral guidewire, Ureteral orifice

## Abstract

**Background:**

En bloc resection of bladder tumor provides improved specimen quality compared with conventional transurethral resection of bladder tumor; however, periorifice tumors remain technically challenging due to the risk of ureteral injury. We reviewed the perioperative and short-term outcomes of patients who underwent guidewire-assisted en bloc resection for periorifice non-muscle-invasive bladder cancer.

**Methods:**

In this retrospective case series, we included patients with non-muscle-invasive bladder cancer located within 1 cm of the ureteral orifice who underwent guidewire-assisted en bloc resection of bladder tumors between May 2024 and October 2025. A guidewire was inserted into the ipsilateral ureter before en bloc laser resection. Perioperative outcomes, pathological findings, postoperative complications, and follow-up data were collected and descriptively analyzed.

**Results:**

Eleven patients who underwent guidewire-assisted ERBT were included in the final analysis. The median operative time was 26 min. No bladder perforation or obturator nerve reflex was observed in any patient. Minor bleeding was observed in three patients. The detrusor muscle was present, and the surgical margins were negative in all specimens. Histopathology revealed Ta disease in nine patients and T1 disease in two patients, with low-grade tumors present in nine cases and high-grade tumors in two. A ureteral stent was placed in one patient. During a median follow-up of 9 months (range: 3–18 months), no tumor recurrence or ureteral obstruction was detected.

**Conclusions:**

In this retrospective case series, patients who underwent guidewire-assisted en bloc resection of bladder tumor had acceptable perioperative outcomes, with no major ureteral complications observed during short-term follow-up. Larger prospective studies are needed to further evaluate its perioperative, functional, and long-term oncologic outcomes.

## Background

Bladder cancer is one of the most common malignancies worldwide, and approximately 75% of patients present with non-muscle-invasive bladder cancer (NMIBC) [1]. Conventional transurethral resection of bladder tumor (TURBT) remains the standard diagnostic and therapeutic approach [2, 3]. However, its piecemeal nature may lead to specimen fragmentation, loss of tissue orientation, and tumor cell dispersion, thereby compromising pathological assessment and oncological control [4].

En bloc resection of bladder tumor (ERBT) has been introduced to overcome these limitations. By removing the tumor together with the adjacent tissue in a single piece, ERBT preserves specimen integrity, improves the accuracy of staging and margin evaluation, and achieves significantly higher detrusor muscle (DM) detection rates than conventional TURBT [5–7]. Recent evidence has shown that ERBT reduces recurrence rates [8], further supporting its role as a high-quality resection technique.

Despite these advantages, ERBT remains technically challenging in certain anatomical locations, particularly near the ureteral orifice, where the risk of ureteral injury often prevents en bloc removal [2, 9]. Consequently, periorifice tumors are commonly resected piecemeal, reflecting the caution of surgeons regarding potential ureteral injury and postoperative complications.

To facilitate en bloc resection in this high-risk region, we used a guidewire-assisted ERBT technique, in which a guidewire is inserted into the ipsilateral ureter to enhance visualization of the ureteral orifice and provide immediate access for stent insertion if required. In this study, we reviewed the perioperative and short-term outcomes of patients who underwent guidewire-assisted ERBT for periorifice NMIBC.

## Methods

### Study design and patient selection

**Fig. 1:**
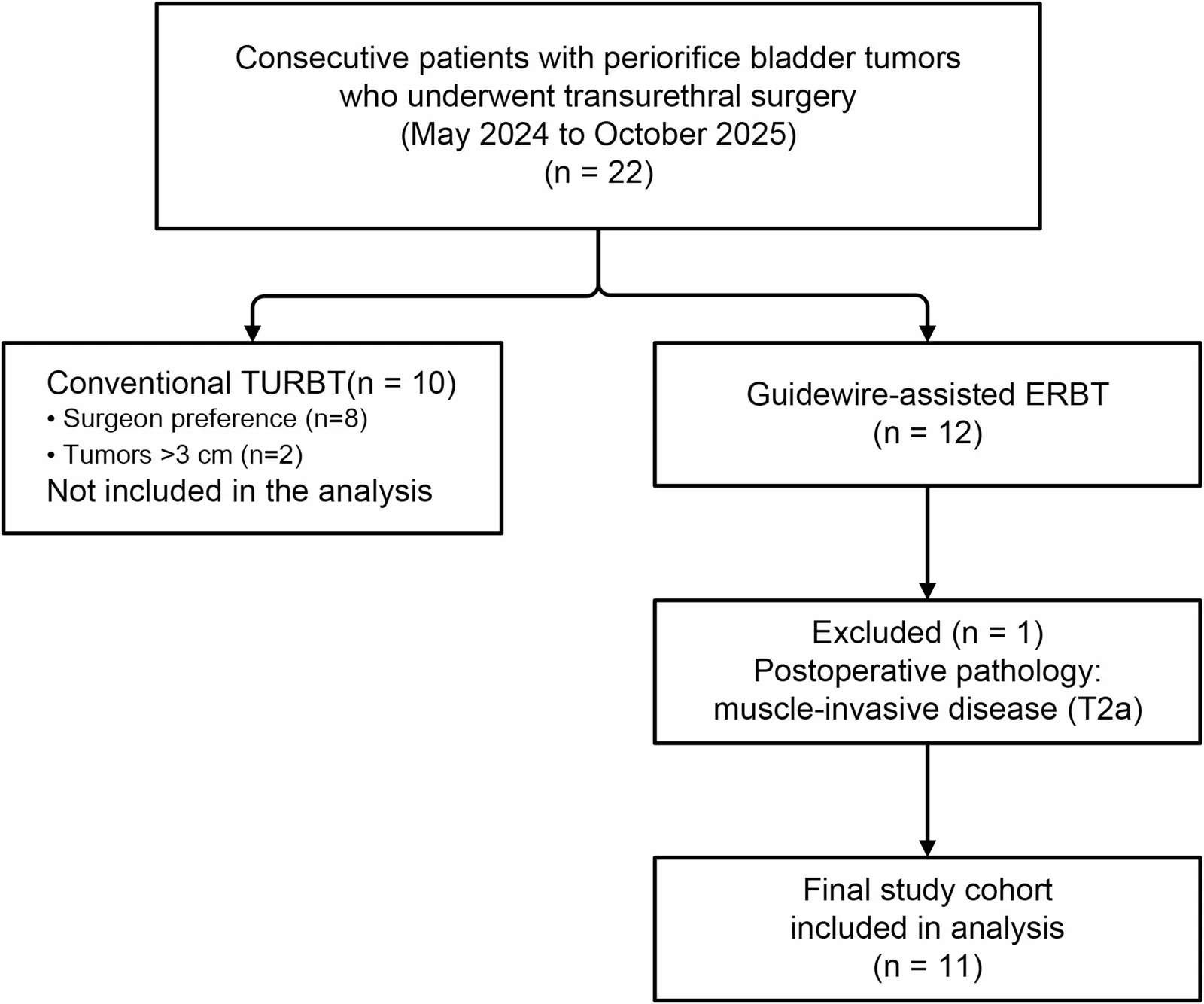
Flowchart of patient identification and selection. TURBT: Transurethral resection of bladder tumor, ERBT: En bloc resection of bladder tumor

In this retrospective case series, we reviewed consecutive patients with periorifice bladder tumors (defined as tumors located within 1 cm of the ureteral orifice) who underwent transurethral surgery between May 2024 and October 2025 at the Second Affiliated Hospital of Soochow University (Fig. 1).

During the study period, 22 patients with periorifice bladder tumors were identified. Complete clinical, pathological, and follow-up data were available for all patients. At our institution, patients with clinically suspected NMIBC, no more than three lesions, and a maximum tumor diameter < 3 cm were generally considered suitable candidates for ERBT. Of the 22 patients, two had tumors > 3 cm and therefore underwent conventional TURBT. Among the remaining 20 patients, 12 underwent guidewire-assisted ERBT, whereas 8 underwent conventional TURBT because they were treated by a surgeon who preferred conventional TURBT.

All guidewire-assisted ERBT procedures were performed by the same experienced surgeon, who had performed more than 100 ERBT procedures before the study period. Among the 12 patients treated with guidewire-assisted ERBT, one was excluded from the final analysis because postoperative histopathology revealed muscle-invasive disease (T2a). Therefore, 11 patients were included in the final cohort. This study was conducted in accordance with the principles of the Declaration of Helsinki and was approved by the Institutional Ethics Committee of the Second Affiliated Hospital of Soochow University (Approval No. JD-HG-2025-139).

As this was a retrospective analysis of anonymized data, the requirement for individual informed consent was waived by the Ethics Committee.

### Surgical technique: guidewire-assisted ERBT

All procedures were performed using a continuous-flow laser resectoscope (F26; Olympus Winter & Ibe GmbH, Hamburg, Germany) and a holmium laser system (DHL, Dahua, Wuxi, China).

**Fig. 2:**
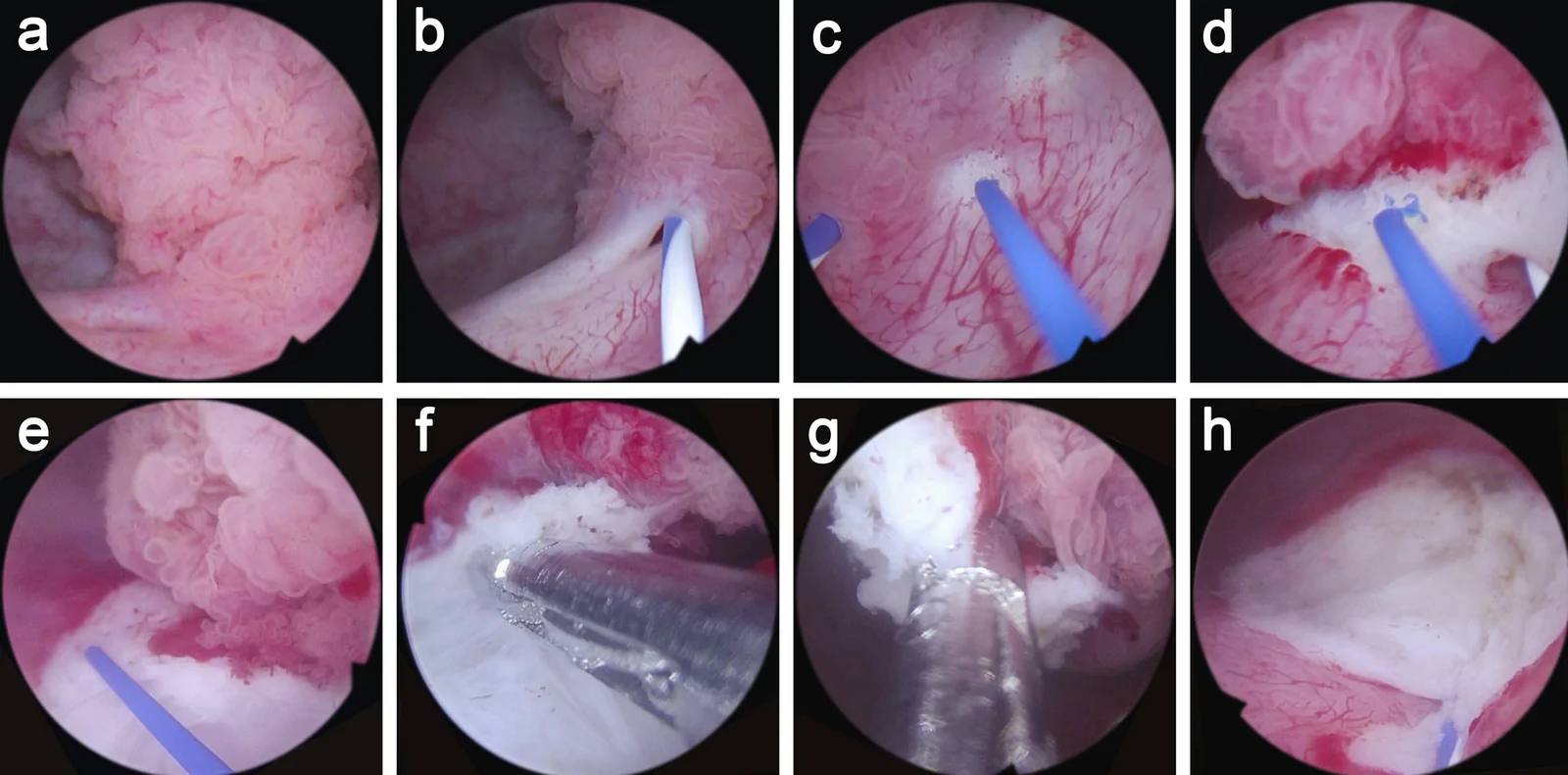
Key steps of guidewire-assisted ERBT near the ureteral orifice. **a** Initial cystoscopic evaluation showing the tumor adjacent to the left ureteral orifice. **b** Insertion of a guidewire into the left ureter. **c **Marking of the lesion margin. **d** Initiation of dissection on the ureteral-orifice side. **e** Sharp dissection within the intramuscular dissection plane of the detrusor muscle. **f **Blunt separation using grasping forceps along the dissection plane. **g** Extraction of the intact specimen through the resectoscope sheath. **h** Post-resection assessment of the ureteral orifice showing partial mucosal injury but preserved anatomical configuration, enabling uncomplicated re-identification; no stent placement was required

After complete cystoscopic examination, with particular attention to the relationship between the tumor base and the ureteral orifice (Fig. 2a), a 0.035-inch × 150-cm straight-tip PTFE-coated striped nitinol guidewire (Boston Scientific, Marlborough, MA, USA) was inserted into the ipsilateral ureter under direct endoscopic visualization through the working channel of the resectoscope, without fluoroscopic guidance or ureteral catheterization (Fig. 2b). The same type of guidewire was used in all cases. After wire placement, the resectoscope was withdrawn, leaving the guidewire in situ. The wire was secured externally to maintain its position. The resectoscope was subsequently reintroduced into the bladder beside the externally fixed guidewire, enabling tumor resection with the wire positioned outside the resectoscope.

The holmium laser was set at 1.0–1.5 J and 20–30 Hz. After marking the tumor margin approximately 5–10 mm from the lesion edge (Fig. 2c), a circumferential incision was made. An adequate depth was confirmed by visualization of the striated DM layer.

Dissection was initiated on the ureteral orifice side (Fig. 2d), making use of the clear visual field at the start of the procedure. The superficial muscle fibers were deliberately transected to enter the intramuscular dissection plane, along which resection then proceeded. Instead of continuous laser cutting, we alternated between laser dissection (Fig. 2e) and blunt separation using grasping forceps (Fig. 2f) to maintain the appropriate dissection plane and facilitate controlled progression of the dissection.

After complete en bloc removal, the specimen was retrieved through the resectoscope sheath (Fig. 2g). A repeat cystoscopic inspection was performed to assess the ureteral orifice (Fig. 2h). Routine random bladder biopsies were not performed. The guidewire was removed at the end of the procedure.

### Pathological evaluation

**Fig. 3:**
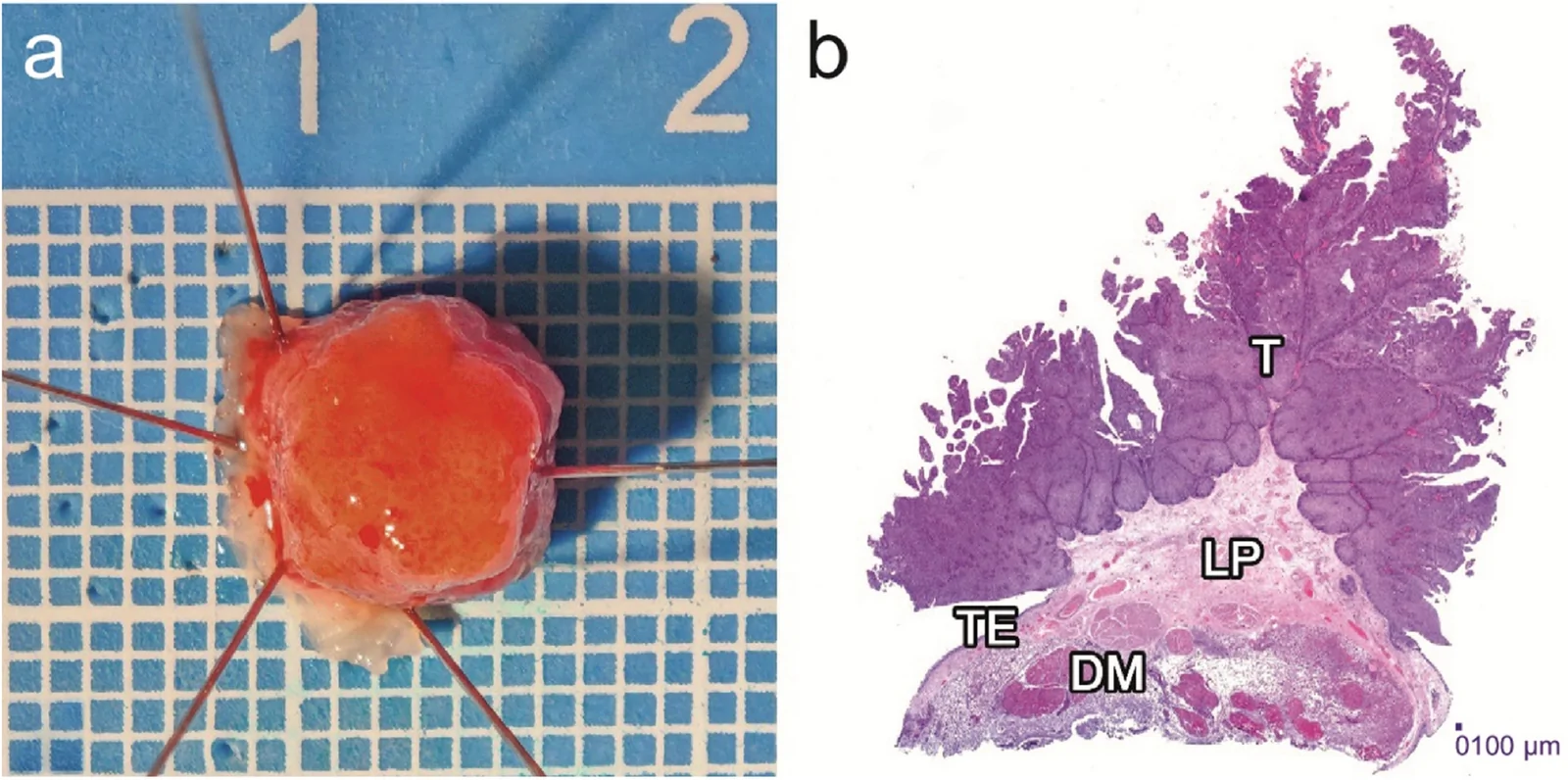
Specimen placement and photomicrograph of a resected lesion. **a** Gross specimen pinned on a board for fixation and orientation. **b** Photomicrograph of a resected tumor (H&E, magnification 20×): T: Tumor, TE: Transitional epithelium, LP: Lamina propria, DM: Detrusor muscle

All specimens were carefully spread and pinned flat on a rubber board (Fig. 3a) before being immersed in 10% neutral buffered formalin for fixation. Subsequently, the specimens were submitted for histopathological evaluation by an experienced genitourinary pathologist. Specimen integrity and orientation were assessed macroscopically prior to sectioning, followed by routine hematoxylin and eosin (H&E) staining for microscopic examination (Fig. 3b). Tumor grade was assigned according to the 2022 World Health Organization classification system. The pathological stage was determined according to the 2017 Tumor–Node–Metastasis (TNM) classification system, based on the deepest level of tumor invasion identified in the specimen. The presence of DM and resection margin status were systematically assessed and recorded.

Risk stratification was performed according to the European Association of Urology (EAU) NMIBC risk classification system, incorporating tumor stage, grade and additional clinical risk factors as defined by the guidelines.

### Perioperative management and follow-up

Perioperative antibiotic prophylaxis was administered to all patients according to the institutional protocol for transurethral procedures. A single intravesical instillation of mitomycin C was administered within 6 h postoperatively. Continuous bladder irrigation was performed overnight. Routine postoperative laboratory assessments, including complete blood count, renal function, and serum electrolytes, were performed on postoperative day 1 in all patients.

Postoperative follow-up included cystoscopic surveillance and assessment of ipsilateral upper urinary tract patency by ultrasonography. Subsequent intravesical therapy was administered according to postoperative risk stratification and EAU guideline recommendations.

### Outcome definitions and complication assessment

Ureteral injury was defined as mechanical or thermal damage involving the intramural or distal ureter that resulted in structural disruption or impairment of ureteral drainage. Ureteral injury was identified based on operative reports, surgical video review, or postoperative imaging findings demonstrating ureteral perforation, laceration, transection, periureteral urinary leakage, or ipsilateral ureteral obstruction requiring drainage or surgical intervention during follow-up.

Ureteral obstruction was defined as new-onset ipsilateral upper urinary tract dilatation with a renal pelvic anteroposterior diameter ≥ 10 mm, or a ≥ 5 mm increase compared with preoperative measurements.

Tumor recurrence was defined as histologically confirmed urothelial carcinoma detected during follow-up cystoscopy.

Perioperative complications were graded according to the Clavien–Dindo classification system.

### Statistical analysis

All statistical analyses were performed using SPSS version 26.0 (IBM Corp., Armonk, NY, USA). Continuous variables are summarized as the median (range) and categorical variables as frequency (percentage). Because of the limited sample size, all analyses were descriptive and exploratory.

## Results

### Patient and tumor characteristics

Of the 12 patients who underwent guidewire-assisted ERBT, one was excluded from the final analysis because postoperative histopathology revealed muscle-invasive disease (T2a). In this excluded case, guidewire placement and en bloc resection were successfully completed without intraoperative conversion or procedure-related complications.

The remaining 11 patients (eight men and three women) with periorifice NMIBC constituted the final study cohort. The median patient age was 61 (range: 41–82) years. Tumors were located on the left side in eight patients and on the right side in three patients. The median tumor size was 11 (range: 8–15) mm. All tumors were solitary, and no patient had concomitant carcinoma in situ. Baseline patient and tumor characteristics are summarized in Table 1. 

**Table 1:** Patient and tumor characteristics

Case	Sex	Age (y)	Lesion number	Side	Size (mm)	Stage	Grade	Risk group
1	F	69	1	Left	12	Ta	Low	Low
2	M	82	1	Left	15	Ta	Low	Low
3	M	41	1	Left	8	Ta	Low	Low
4	F	75	1	Right	13	T1	High	High
5	F	62	1	Left	9	Ta	Low	Low
6	M	43	1	Right	10	Ta	Low	Low
7	M	47	1	Left	12	Ta	High	Intermediate
8	M	69	1	Left	8	Ta	Low	Low
9	M	54	1	Left	9	Ta	Low	Low
10	M	56	1	Left	15	T1	Low	Low
11	M	61	1	Right	11	Ta	Low	Low

*Abbreviations*: *y* years

### Perioperative outcomes

The perioperative outcomes of the 11 included patients are summarized in Table 2. The median operative time was 26 min (range, 15–37 min). Minor bleeding (Clavien–Dindo Grade I) occurred in three patients and was effectively controlled using laser coagulation. No obturator nerve reflex or bladder perforation was observed. The Foley catheter was removed at a median of 1 day postoperatively (range, 1–2 days). The median postoperative length of stay was 2 days (range, 2–3 days). No postoperative urinary tract infections, blood transfusions, or perioperative readmissions occurred. A ureteral stent was placed in one patient because marked architectural disruption of the ureteral orifice was observed, and re-identification of the orifice after guidewire removal was anticipated to be difficult. The stent was removed 2 weeks later, and follow-up ultrasonography 2 weeks after stent removal showed no hydronephrosis.

**Table 2:** Operative details and postoperative outcomes

Case	PT (min)	AE	DM	CM	Stent	HN	FP	CD (d)	Postoperative LOS (d)	FU (mo)	Rec
1	23	None	Yes	Yes	No	No	No	1	2	18	No
2	30	MB	Yes	Yes	No	No	Yes	2	3	16	No
3	15	None	Yes	Yes	No	No	No	1	2	13	No
4	37	MB, OI	Yes	Yes	Yes	No	No	2	3	12	No
5	26	None	Yes	Yes	No	No	No	1	2	9	No
6	28	None	Yes	Yes	No	No	No	2	3	9	No
7	34	MB	Yes	Yes	No	No	No	2	3	8	No
8	20	None	Yes	Yes	No	No	No	1	2	7	No
9	17	None	Yes	Yes	No	No	No	1	2	6	No
10	32	None	Yes	Yes	No	No	No	2	3	5	No
11	23	None	Yes	Yes	No	No	No	1	2	3	No

*Abbreviations*: *AE* adverse events, *CD* catheter duration, *CM* clear margin, *DM *detrusor muscle, *FP* flank pain, *FU *follow-up, *HN* hydronephrosis, *LOS* length of stay, *MB* minor bleeding, *OI* orifice injury, *PT* procedure time, *Rec* recurrence

Median preoperative and postoperative day 1 hemoglobin levels were 147 g/L (range, 111–168) and 142 g/L (range, 105–164), respectively, while the corresponding serum creatinine values were 80 μmol/L (range, 46–124) and 82 μmol/L (range, 52–130), respectively. Transient irritative urinary symptoms, including frequency, urgency, and dysuria, were documented in five patients after catheter removal; none required additional treatment. 

### Histopathological findings

Histopathological findings showed that the tumor grade was low in nine patients and high in two, and the pathological stage was Ta in nine patients and T1 in two. The DM was present in all specimens, thereby confirming an adequate resection depth; all surgical margins were negative. 

### Adjuvant therapy

All patients received a single immediate postoperative intravesical instillation of mitomycin C. Subsequent adjuvant therapy was administered according to EAU risk stratification. Nine patients were classified as low risk and received no further intravesical therapy. One patient with intermediate-risk disease received induction intravesical mitomycin C followed by maintenance therapy. One patient with high-risk disease received intravesical BCG induction therapy followed by maintenance therapy. 

### Follow-up results

he median follow-up duration was 9 months (interquartile range, 6–13 months; range, 3–18 months). Follow-up data were available for all patients. Four patients (36.4%) had a follow-up duration of ≥ 12 months. One patient developed ipsilateral flank pain on postoperative day 1. Ultrasonography showed renal pelvic dilatation measuring 8 mm in the anteroposterior diameter. The pain resolved with antispasmodic and analgesic therapy. No ureteral stent or percutaneous drainage was required. Repeat ultrasonography at 1 and 2 weeks confirmed complete resolution of pelvic dilatation without hydronephrosis.

All patients underwent cystoscopic surveillance at 3 months postoperatively. Subsequent surveillance was performed according to risk stratification, with the 10 patients with low- or intermediate-risk disease undergoing cystoscopic follow-up at 6-month intervals and the patient with high-risk disease undergoing cystoscopic surveillance and urinary cytology every 3 months.

Ultrasonography was performed in all patients at 2 weeks and 3 months postoperatively and subsequently before each cystoscopic surveillance visit. No ureteral obstruction was identified, and no patient underwent computed tomography urography or other cross-sectional imaging. 

## Discussion

This study reports the perioperative and short-term follow-up findings of patients who underwent guidewire-assisted en bloc resection for periorifice NMIBC. ERBT has shown high feasibility and technical success in the management of NMIBC [4, 6]. However, periorifice tumors remain technically challenging because their anatomical proximity to the intramural ureter increases the risk of thermal or mechanical injury [10–12]. Consequently, many surgeons continue to favor piecemeal TURBT in this region, despite its well-recognized limitations in pathological assessment.

One potential advantage of guidewire-assisted ERBT is that the guidewire may facilitate continuous identification of the ureteral orifice throughout the procedure. Although temporary ureteral stent placement was required in one patient because of marked disruption of the ureteral orifice, no hydronephrosis or evidence of ureteral obstruction was observed during follow-up.

In our technique, dissection was initiated from the ureteral orifice side while the visual field remained optimal at the beginning of the procedure. This sequencing was intended to minimize blind manipulation near the intramural ureter and to allow controlled separation of the tumor base under direct visualization. We emphasize that this represents a technical preference based on intraoperative considerations rather than a strategy supported by comparative evidence.

Postoperative distal ureteral obstruction after tumor resection involving the ureteral orifice has been reported in 4%–16% of cases [10, 13]. However, whether prophylactic stenting reduces this risk remains controversial. Mano et al. showed that orifice resection without stenting rarely led to significant obstruction when performed with judicious energy settings [10]. Similarly, Faba et al. reported no clear advantage of routine stenting and recommended selective placement only when deep or extensive orifice resections occurred [14]. Conversely, Chou et al. showed higher stenosis rates in markedly damaged orifices, indicating the potential protective role of stenting [11].

Recent studies have suggested that ureteral stents may predispose patients to metachronous upper tract urothelial carcinoma (UTUC), thereby indicating that unnecessary stent placement should be avoided [15, 16]. Collectively, these findings support a selective rather than routine approach.

Migliari et al. demonstrated that inserting a ureteral guidewire prior to thulium laser ERBT facilitates resection of tumors located at or near the orifice without the need for routine stenting [17]. Similar to the safety wire concept in ureteroscopy, the guidewire provides an anatomical reference and ensures immediate access for stent insertion if required [18]. Although alternative strategies such as visual landmark-guided dissection, circumferential marking, or ureteral catheterization may also facilitate identification of the ureteral orifice, the guidewire combines both visual guidance and the ability to facilitate subsequent stent placement when necessary. Our findings align with this rationale.

When upper tract decompression is required, ureteral stents and percutaneous nephrostomies have comparable risks of metachronous UTUC [15, 19]. Considering that percutaneous nephrostomy is more invasive and carries a higher procedure-related morbidity, internal stenting is generally preferred when feasible [20]. These considerations support a selective rather than routine approach to ureteral stenting. In our series, a ureteral stent was placed in only one patient because marked disruption of the ureteral orifice was observed and subsequent identification of the orifice was anticipated to be difficult.

Energy sources play an important role in minimizing ureteral injury. The coagulation current near the orifice has been linked to deeper thermal penetration and subsequent fibrosis, whereas a pure cutting current or laser energy produces more superficial effects with reduced collateral damage [2, 21]. Holmium and thulium laser ERBT have been reported to provide precise cutting with shallow penetration, making them suitable for delicate areas such as the ureterovesical junction [17, 22]. In our series, all procedures were performed using a holmium laser, which may have contributed to the favorable outcome with no postoperative ureteral obstruction.

As transurethral surgery lacks dynamic traction, several operative maneuvers may help overcome restricted exposure in the periorifice region.

1. Guidewire-assisted retraction. By adjusting the relative position of the resectoscope and the externally fixed guidewire, gentle retraction of the ureteral orifice can be achieved by increasing the working distance and improving the exposure of the tumor–orifice interface.
2. Hybrid sharp–blunt dissection. Alternating holmium laser cutting with blunt separation using forceps helps maintain the natural dissection plane and prevents unintentional deep cutting; this approach is supported by evidence highlighting the role of combined dissection techniques in maintaining the anatomical planes [23–25].
3. Hydrodynamic “traction” through irrigation. Continuous-flow systems are known to assist visualization by clearing blood and debris [4, 8]. We observed that increasing the irrigation flow could lift the partially dissected tissue, exposing the underlying plane without disrupting tissue integrity, an effect analogous to temporary traction.
4. Sheath-assisted elevation. In larger lesions, advancing the sheath gently after partial basal release may elevate the tumor and improve the visualization of residual attachments.

Although none of these maneuvers replicate the dynamic, multidirectional traction achievable in open or laparoscopic surgery, they provide practical strategies within the current endoscopic limitations. Emerging technologies, including rotatable bi-channel systems [26, 27] and early transurethral robotic platforms [28], may further enhance traction and facilitate more controlled dissection in such anatomically demanding regions.

This study has several limitations. First, the sample size was small, and all procedures were performed by a single experienced surgeon at a single center, which may limit generalizability and introduce operator-related bias. Second, the retrospective design and absence of a control group preclude causal attribution of the observed outcomes to the guidewire-assisted modification of ERBT. The absence of ureteral complications, negative resection margins, and DM presence may have been influenced by surgeon expertise or patient selection rather than the surgical modification itself.

Additionally, this study included a highly selected patient population. International consensus statements and published ERBT literature generally recommend ERBT for patients with a limited tumor burden, including tumors ≤3 cm and no more than three lesions [4, 9, 29]. The patients included in the present series represented an even more selected subgroup, as all tumors were solitary, relatively small (8–15 mm in maximum diameter), and none completely overlaid or obscured the ureteral orifice, a scenario in which guidewire placement would likely be technically infeasible. Therefore, these findings may not be generalizable to more complex periorifice tumors.

All patients received immediate postoperative intravesical chemotherapy, and most were classified as low risk. Thus, the absence of recurrence may reflect tumor biology and guideline-based adjuvant therapy rather than the surgical technique alone.

Finally, follow-up was relatively short, and only a small proportion of patients had follow-up beyond 12 months. Accordingly, no firm conclusions regarding long-term oncologic or ureter-related functional outcomes, particularly ureteral obstruction rates, can be drawn. Postoperative renal function and urinary symptoms were not systematically assessed using standardized measures. Larger prospective, controlled, multicenter studies with longer follow-up are needed to validate these findings. 

## Conclusions

In this retrospective case series, patients who underwent guidewire-assisted en bloc resection of bladder tumor had acceptable perioperative outcomes, with no major ureteral complications observed during short-term follow-up. Larger prospective comparative studies are needed to further evaluate its perioperative, functional, and long-term oncologic outcomes.

## Data Availability

The data analyzed in this study are available from the corresponding author upon reasonable request.

## Abbreviations

AE: Adverse events
BCG: Bacillus Calmette–Guérin
CD: Catheter duration
CM: Clear margin
DM: Detrusor muscle
EAU: European Association of Urology
ERBT: En bloc resection of bladder tumors
FP: Fank pain
FU: Follow-up
H&E: Hematoxylin and eosin
HN: Hydronephrosis
LOS: Length of stay
MB: Minor bleeding
NMIBC: Non-muscle-invasive bladder cancer
OI: Orifice injury
PT: Procedure time
Rec: Recurrence
TURBT: Transurethral resection of bladder tumors
UTUC: Upper tract urothelial carcinoma
